# *Saprochaete clavata* Outbreak Infecting Cancer Center through Dishwasher 

**DOI:** 10.3201/eid2609.200341

**Published:** 2020-09

**Authors:** Estelle Menu, Alexis Criscuolo, Marie Desnos-Ollivier, Carole Cassagne, Evelyne D’Incan, Sabine Furst, Stéphane Ranque, Pierre Berger, Françoise Dromer

**Affiliations:** Institut Hospitalo-Universitaire, Méditerranée Infection, Marseille, France (E. Menu, C. Cassagne, S. Ranque);; Institut Pasteur, Paris, France (A. Criscuolo, M. Desnos-Ollivier, F. Dromer);; Centre de Lutte Contre le Cancer, Institut Paoli-Calmettes, Fédération Unicancer, Marseille (E. D’Incan, S. Furst, P. Berger)

**Keywords:** acute myeloid leukemia, France, fungemia, fungi, genome sequencing, hematological malignancies, pathogenic yeasts, phylogenetic clades, Saprochaete clavata

## Abstract

*Saprochaete clavata* is a pathogenic yeast responsible for rare outbreaks involving immunocompromised patients, especially those with hematologic malignancies. During February 2016–December 2017, we diagnosed *S. clavata* infections in 9 patients (8 with fungemia), including 3 within 1 month, at a cancer center in Marseille, France. The patients (median age 58 years), 4 of 9 of whom had acute myeloid leukemia, were hospitalized in 3 different wards. Ten environmental samples, including from 2 dishwashers and 4 pitchers, grew *S. clavata*, but no contaminated food was discovered. The outbreak ended after contaminated utensils and appliances were discarded. Whole-genome sequencing analysis demonstrated that all clinical and environmental isolates belonged to the same phylogenetic clade, which was unrelated to clades from previous *S. clavata* outbreaks in France. We identified a dishwasher with a deficient heating system as the vector of contamination.

*Saprochaete clavata* (previously *Geotrichum clavatum*) is a rare emerging pathogen, an ascomycetous yeast-producing arthroconidia that causes invasive fungal infections in immunocompromised patients. The species has mainly been reported in Europe, often associated with sporadic cases or small outbreaks (*1*,*2*). Unlike *Magnusiomyces capitatus* (*3*,*4*), which has been associated with dairy products, *S. clavata* has rarely been isolated from environmental samples (*5*,*6*). Patients most at risk for infections from *Geotrichum* spp. have hematologic diseases with severe neutropenia (*7*) and are undergoing chemotherapy, mainly with cytarabine (*1*) or caspofungin (*8*). They often have central venous catheters (*9*). 

In recent years, *S. clavata* fungemia outbreaks associated with high mortality rates in vulnerable patients with malignancies have been described throughout Europe, mainly in France (*1*), Italy (*2*,*10*), Czechia (*11*), and Spain (*12*). No source of contamination was identified in any of these outbreaks despite thorough investigation.

During February 2016–December 2017, the Paoli-Calmettes Institute, a cancer center in Marseille, France, was faced with an outbreak of *S. clavata* infections involving 9 patients hospitalized in 3 different wards, suggesting a common source of contamination. We describe the findings of an outbreak investigation that recovered *S. clavata* in different environmental samples, including from a dishwasher in the central kitchen and another, available to patients and their families, in the stem-cell transplant ward. Whole-genome sequencing (WGS) confirmed that the environmental and clinical isolates from patients belonged to the same phylogenetic clade. Handwashing, avoiding direct skin contact, checking air quality, and sterilizing food are routine practice to prevent contamination in hematology wards; however, examining dishwashers for contamination and operability may not be done routinely. Our findings should prompt adding dishwasher inspections to guidelines for preventing infection. 

## Materials and Methods

### Case Definition Criteria

We defined *S. clavata* infection by obtaining ≥1 positive results for *S. clavata* blood culture from a usually sterile body site or from a bronchoalveolar lavage or tracheal aspirate of the respiratory tract. Infection was also confirmed by observing pleural fluid in a patient with pleural effusion or lung infection.

### Mycologic Investigation

We collected a rectal swab specimen from all patients hospitalized in the stem-cell transplant unit during December 20–30, 2017. In addition, during December 22, 2017–January 19, 2018, we collected 95 environmental samples from food (powdered milk, a pea-sized amount from each package of cheese); tap water in 2 patients’ room and water used for the coffee machine in 1 kitchen (500 µL); air filters; food-contact surfaces; non–food-contact surfaces in the rooms of infected patients; various kitchenware (vacuum flasks, cutlery); tables and chairs in the ward’s kitchen; and microwaves, refrigerators, and dishwashers, including the dishwasher in the ward’s central kitchen. For the dishwashers, we sampled inner surfaces, door seals, and the water outlet. 

We used Sigma Transwab MW176S MWE medical wire sterile dry cotton swabs (Sigma Transwab, https://www.mwe.co.uk) for sampling as wide an area as possible. We discharged swabs in liquid Amies medium, then streak-plated the samples on Sabouraud dextrose agar plates supplemented with gentamicin and chloramphenicol (Bio-Rad, https://www.bio-rad.com) and BBL CHROMagar Candida plate (BD, https://www.bd.com). We identified species using Bruker Biotyper version MBT 3.1 matrix-assisted laser desorption/ionization time-of-flight (MALDI-TOF) mass spectrometry (Bruker, https://www.bruker.com) and nucleotide sequence analysis of the internal transcribed spacer (ITS) regions of the rRNA gene, as described elsewhere (*13*). The ITS sequences of the isolates were compared to those of the *S. clavata* type strain CBS425.71 (GenBank accession no. KF984489) isolated in Baltimore, Maryland, USA, in 1971. 

All the strains we recovered from environmental and clinical samples and identified as *S. clavata* were stored at −20°C in cryotubes with bead tune Cryosystème Protect (Dutscher, https://www.dutscher.com). After subculturing all of the samples on Sabouraud agar slant (Bio-Rad), we sent them to the French National Reference Center for Invasive Mycoses and Antifungals (Institut Pasteur, Paris, France) for further characterization and comparison with selected clinical isolates collected through the nationwide surveillance program (Appendix Table). 

### WGS

After checking purity on chromogenic medium, we extracted DNA using a NucleoMag Plant kit (Macherey-Nagel, https://www.mn-net.com) in a KingFisher Flex system (Thermo Fisher Scientific, https://www.thermofisher.com). We sequenced whole genomes from each selected isolate (17 clinical and 10 environmental isolates) at the Mutualized Platform for Microbiology (Institut Pasteur, Paris, France) using a NextSeq 500 sequencer (Illumina, https://www.illumina.com). We constructed libraries using Nextera XT technology (Illumina) and sequenced genomes using a 2 × 150 nt paired-end run strategy. We preprocessed all reads with AlienTrimmer version 0.4.0 (https://bioweb.pasteur.fr/packages/pack@AlienTrimmer@0.4.0) to remove exogenous or low-quality bases, leading to a mean of 8.47M paired-end reads per sample (≈140 × sequencing depth, mean). We deposited FASTQ files for all isolates from Marseille at the European Nucleotide Archive BioProject (accession no. PRJEB36345). 

### Phylogenetic Analysis 

For phylogenetic comparison, we used WGS data from 10 isolates studied during an outbreak described by Vaux et al. (BioProject accession no. ERP003645) (*1*); all reads from the BioProject ERP003645 isolates were preprocessed as described in previous sections. (The patients from whom the cultures were isolated correspond to patients 11–20 in the Appendix Table.) These reads included 5 isolates from epidemic clade A (CNRMA12.494, CNRMA12.559, CNRMA12.637, CNRMA12.667, CNRMA12.647) and 5 from epidemic clade B (CNRMA8.1167, CNRMA11.1183, CNRMA12.304, CNRMA12.615, CNRMA12.634). Overall, we studied a total of 38 isolates: 10 from BioProject ERP003645; 26 clinical and environmental isolates recovered in Marseille during the outbreak or its investigation, plus 1 clinical isolate, CNRMA15.181, recovered in 2015 in the same hospital in Marseille; and the *S. clavata* strain (CBS425.71). 

For each preprocessed read sample, we performed short read mapping against the genome sequence of *S. clavata* clade A isolate CNRMA12.647 (GenBank accession no. CBXB000000000.1) using minimap2 version 2.17-r941 (*14*). We then inferred a pseudogenome following 4 rules: 1) we considered only aligned reads and sequenced bases associated with a Phred score >20; 2) we replaced each position with the character states observed in >80% of the aligned residues at that position; 3) we replaced every position covered by <10 aligned reads with the unknown character state “?”; and 4) we replaced all polymorphic positions located within strand-biased (set as <5 aligned reads on ≥1 strand) or over-covered regions (set as >200×) with the character state “X.” Finally, after pooling all pseudogenome sequences into a unique matrix of aligned nucleotide characters, we discarded each position containing >10% undefined character states (?, –, X, or N), resulting in 12,053,164 characters (including 261 variable characters), which we used to infer a maximum likelihood phylogenetic tree using IQ-TREE (http://www.iqtree.org) (*15*). To approximate the number of single-nucleotide polymorphisms (SNPs) shared by each branch of the phylogenetic tree, each branch length was multiplied by the total number of analyzed characters (i.e., 12,053,164) and the result was rounded to the closest integer. 

### Growth Temperature Testing

We analyzed the ability of 3 isolates of *S. clavata* (CBS425.71 type strain, CNRMA15.100, CNRMA14.292) and 3 isolates of *M. capitatus* (CBS162.80 type strain, CNRMA17.803, CNRMA17.775) to grow at high temperatures after 48 and 72 h of incubation. We subcultured isolates on Sabouraud agar medium at 30°C for 48h, then plated suspensions containing 10^3^, 10^2^, 10, and 1 colony-forming units in 5 µL of sterile distilled water on Sabouraud agar plates and incubated samples of each concentration at 30°C, 35°C, 37°C, 40°C, 45°C, and 48°C. 

## Results

### Characteristics of the Patients 

**Figure 1:**
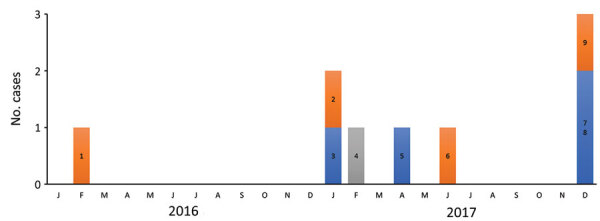
Timeline of outbreak for 9 cases of infection with *Saprochaete clavata* identified in a single center at the Institut Paoli-Calmettes, Marseille, France, February 2016–December 2017. The patients were hospitalized in 3 wards: the hematology unit (orange bar sections), the stem cell transplant unit (blue bar sections), and the intensive care unit (gray bar sections). Numbers 1–9 correspond to patient numbers in the Table.

In December 2017, *S. clavata* infections were diagnosed in 3 patients (numbers 7–9 in the Table) within 3 weeks of admission to the Paoli-Calmettes Institute. This timing suggested a common source of contamination, even though the patients were hospitalized in 2 different wards, the stem-cell transplant and hematology units. A retrospective review of laboratory files revealed that *S. clavata* infection had been diagnosed in 6 additional patients during February 2016–July 2017 (Table; Figure 1), bringing the total identified to 9 patients. The 6 patients found retrospectively had been hospitalized in 3 different wards, the stem-cell transplant, hematology, and intensive care units. The median age of the 9 patients was 58 years (range 38–68 years); 6 (67%) were male. All of the patients had central venous catheters; 4 (44%) were treated for acute myeloid leukemia and 6 (67%) had cytarabine chemotherapy. Of the 41 samples testing positive for *S. clavata*, 35 (85%) were blood cultures; fungemia was detected in 8 (89%) of 9 patients on the basis of a mean of 4 (range 1–9) blood samples positive for *S. clavata*. In 5 patients, results were positive only for the blood samples. Results from rectal swab cultures were positive only for patient 8 (Table). Of note, 5 patients had digestive symptoms. The 90-day case fatality rate was 55% (5/9); median survival time for those 5 patients was 7 days after the first positive culture. 

**Table:** Characteristics of patients with a culture positive for *Saprochaete clavata* in Marseille, France, February 2016–December 2017*

Characteristic	**Patient no.**								
	1	2	3	4	5	6	7	8	9
Age, y	58	38	45	66	57	68	65	56	68
Sex	M	F	M	F	M	M	F	M	M
Hospitalization ward	H	H	T	ICU	T	H	T	T	H
Immune status									
Underlying disease	Lymphoma	AML	MDS	Lymphoma	CLL	AML	ALL	AML	AML
Lymphocyte count, G/L	<0.1	0.1	5.6	0.2	0.1	0.1	0.8	0.1	0.1
Severe neutropenia, <500 /mm^3^	Yes	Yes	No	Yes	Yes	Yes	Yes	Yes	Yes
Duration of neutropenia at time of positive culture, d	6	51	0	4	36	27	0	21	21
BMT	Yes	No BMT	Yes	No BMT	Yes	No BMT	Yes	Yes	Yes
Days from BMT to first positive culture	9		90		75		61	3	>90
Clinical signs at the time of positive culture									
Fever, temperature >38°C	Yes	Yes	Yes	Yes	NA	Yes	NA	Yes	Yes
Digestive symptoms	Yes	Yes	NA	NA	NA	NA	Yes	Yes	Yes
Diarrhea	Yes		NA	NA	NA	NA	Yes	Yes	
Constipation	NA	Yes	NA	NA	NA	NA		NA	Yes
Pulmonary symptoms	NA	Yes	Yes	NA	NA	NA	Yes	NA	Yes
Skin lesions	NA	NA	NA	Yes	Yes	NA	Yes	NA	NA
Positive culture results									
Date of first positive culture	2016 Feb 3	2017 Jan 16	2017 Jan 18	2017 Feb 26	2017 Apr 17	2017 Jun 29	2017 Dec 5	2017 Dec 10	2017 Dec 29
Days after admission	16	51	6	14	80	27	68	20	21
No. positive samples	1	1	2	7	5	9	1	10	5
Blood	1	1	None	5	4	9	1	9	5
Respiratory tract	None	None	2	2	1	None	None	None	None
Stool, rectal swab	None	None	None	None	None	None	None	1	None
Outcome									
Death within 90 d	No	Yes	Yes	Yes	No	Yes	Yes	No	No
Days after first positive culture	DNA	12	57	7	DNA	4	6	DNA	DNA
Treatment									
Venous access	Yes	Yes	Yes	Yes	Yes	Yes	Yes	Yes	Yes
Echinocandins	Micafungin	NP	NP	NP	NP	NP	Caspo	NP	NP
Azoles	NP	PCZ	NP	NP	VCZ	PCZ	VCZ	VCZ	VCZ, PCZ
Cytarabine	Yes	Yes	NP	NP		Yes	Yes	Yes	Yes
Ibrutinib	NP		NP	NP	Yes				
Apheresis platelet concentrates	NP	Yes	NP	NP	Yes	Yes	Yes	Yes	Yes
*ALL, acute lymphoblastic leukemia; ANL, acute myeloid leukemia; BMT, bone marrow transplant; Caspo, caspofungin; CLL, chronic lymphocytic leukemia; DNA, does not apply; H, hematology; ICU, intensive care unit; MDS, myelodysplastic syndromes; NA, not available; NP, not prescribed; PCZ, posaconazole; T, stem-cell transplant; VCZ, voriconazole. †Bronchoalveolar lavage, tracheal aspirate.									

### Mycological and Environmental Investigation

**Figure 2:**
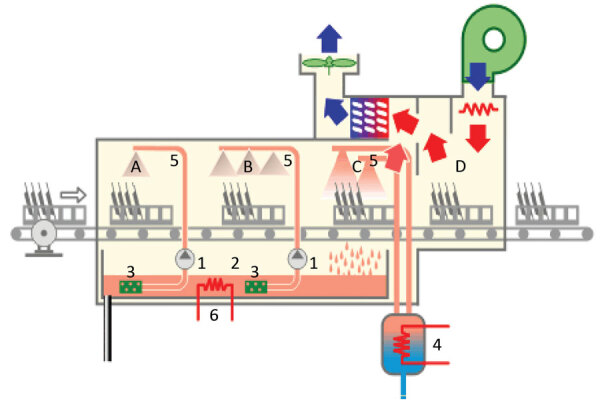
Schematic of dishwasher implicated in outbreak of *Saprochaete clavata* at the Institut Paoli-Calmettes, Marseille, France, February 2016–December 2017. A) Prewash area; B) wash area; C) rinse area; D) drying area. 1, pump; 2, prewash and wash trays; 3, filters; 4, rinse water heater; 5, wash arm; 6, wash heat resistor. Blue arrows indicate cool air flow; red arrows indicate hot air flow. (Figure modified from https://energieplus-lesite.be/techniques/cuisine-collective6/laverie-vaisselle/lave-vaisselle-description [cited 2020 May 20].)

Among the 95 environmental samples, 75 were sterile, and 10 tested positive for fungi other than *S. clavata* (*Penicillium rubens*, *Lecytophora* sp., *Aspergillus creber*, *Alternaria citri*, *Trichoderma viride*, *Exophiala dermatidis*, *Alternaria alternata*, *Candida lusitaniae*, *Candida parapsilosis*, *Scopulariopsis cinerea*, and *Geotrichum capitatum*). Of the 10 *S. clavata*–positive samples, we collected 6 from the kitchen in the stem-cell transplant ward: 4 samples from the dishwasher (water outlet, interior surfaces, and door seal) and 2 samples from vacuum flasks, 1 each used for coffee and milk. Two of those samples had additional fungi species: milk recovered in 2015 in a patient pitcher lid in the hematologic ward contaminated with *C. lusitaniae* and a coffee pitcher lid from the stem cell transplant ward contaminated with *C. lusitaniae* and *C. parapsilosis*. In the stem cell transplant ward, only a sample from a table surface in patient 8’s room tested positive for *S. clavata*. In the hematology ward, we collected *S. clavata*–positive samples from the coffee and milk pitcher lids but found no contamination of the dishwasher. In the central kitchen dishwasher, samples from the prewash area (Figure 2), where water is sprayed to loosen food particles on the dishes, tested positive for *S. clavata*. Finally, samples from 2 different cheeses, proposed as possible vectors at the time of the outbreak, tested negative. 

### Study of Growth Temperature

The isolates of *S. clavata* and *M. capitatus* tested exhibited similar growth at various temperatures. No growth was detected at ≥48°C. 

### WGS

**Figure 3:**
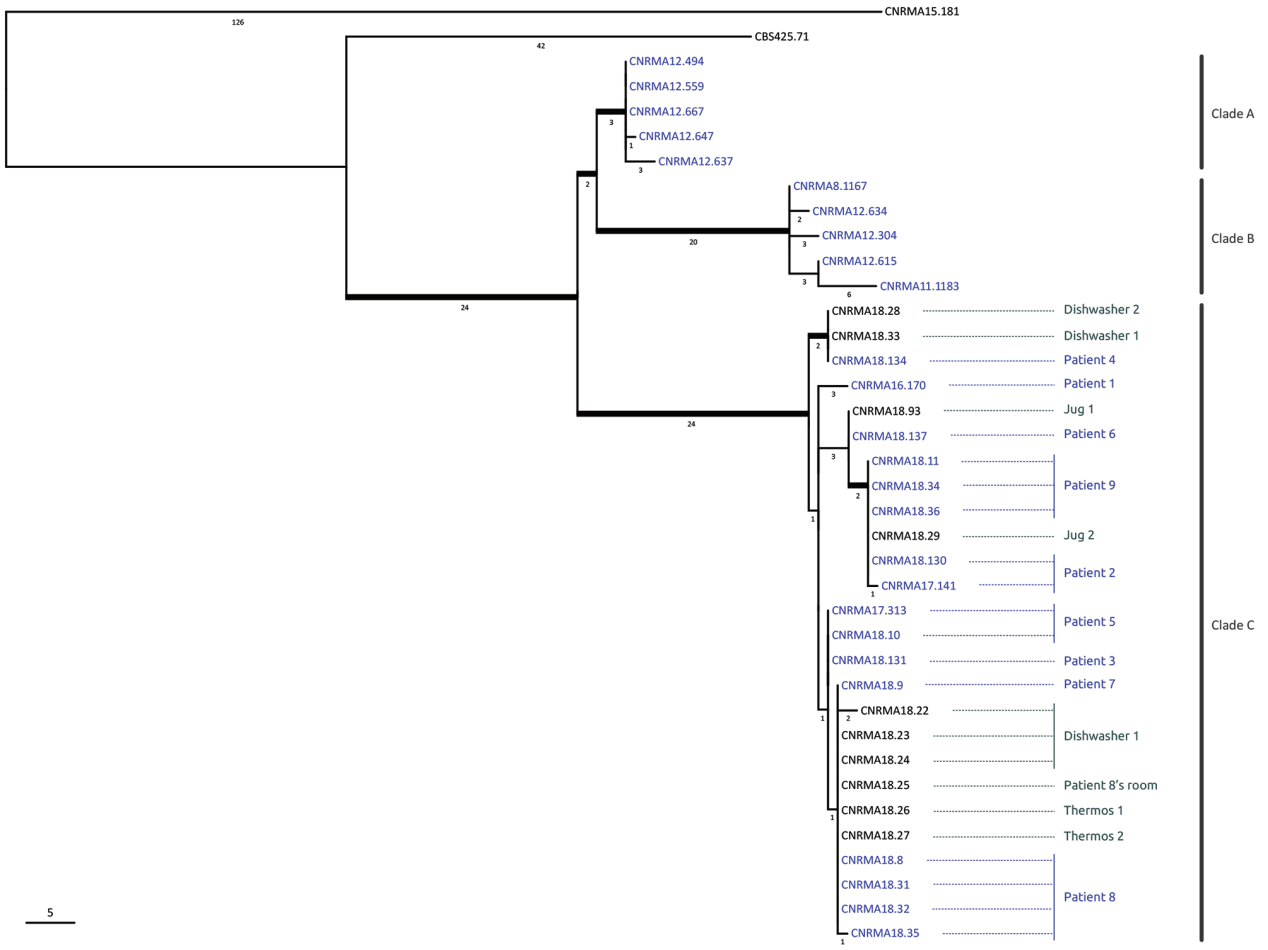
Phylogenetic tree of 38 *Saprochaete clavata* isolates, including isolates from outbreak of *Saprochaete clavata* at the Institut Paoli-Calmettes, Marseille, France, February 2016–December 2017. The unrooted maximum-likelihood tree was inferred from 12,053,164 nt characters with evolutionary model HKY (Hasegawa, Kishino, and Yano, 1985) + FO (base frequencies optimized by ML) + I (proportion of invariable sites optimized by ML). Thick branches are supported by >70% bootstrap supports (500 replicates). The approximated number of single-nucleotide polymorphisms is indicated below each branch. Blue indicates clinical isolates; gray indicates nonclinical isolates. Patient numbers correspond to those in the Table; clades A, B, and C are indicated at the right. Scale bar indicates single-nucleotide polymorphisms.

Bioinformatic analysis of the WGS data yielded a robust phylogenetic classification for 38 isolates (Figure 3). The 5 isolates belonging to clade A and the 5 isolates from clade B (isolation years 2008–2012) clustered in 2 distinct clades, as described elsewhere (*1*). All of the isolates collected in Marseille after February 2016 clustered into a third new monophyletic clade, referred to as clade C, and had an estimated ˂10 SNP difference. Multiple isolates recovered from patients 2, 5, 8, and 9 exhibited ≤1 SNP mean difference. Isolates from both environmental and clinical samples clustered in clade C, suggesting a clonal outbreak with a probable common source. The CNRMA15.181 isolate, which was recovered at the same center in January 2015, clustered in neither clade C nor in any other previously identified clade. 

### Interventions and Control Measures

**Figure 4:**
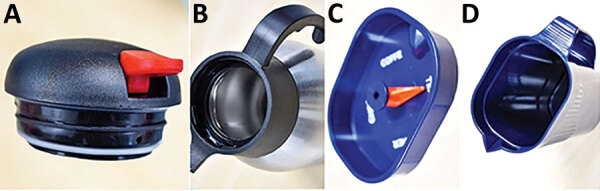
Vacuum flask styles used before and after outbreak of *Saprochaete clavata* at the Institut Paoli-Calmettes, Marseille, France, February 2016–December 2017. A) Old seal; B) old vacuum flask; C) new seal; D) new vacuum flask. The newer model is fully accessible to washing.

We discarded and replaced all *S. clavata*–contaminated fomites and the ward’s dishwasher as soon as contamination was determined. Even if the water temperature could have achieved ˃60°C, the dishwasher was discarded because of incomplete drain cycles, seals in poor condition, and overall aging. We discarded the old vacuum flasks and replaced them with simpler models in which the entire device is accessible to washing (Figure 4). In addition, we instituted mandatory guidelines for thorough cleaning and washing after each use. 

## Discussion 

Small outbreaks and sporadic cases of invasive infections due to *Geotrichum* spp. have been reported mostly, but not exclusively, in Europe. As in this outbreak, patients infected by *M. capitatus* and *S. clavata* often share a common clinical background of severe hematologic malignancy and neutropenia. *M. capitatus* (previously known as *G. capitatum*) is the most common reported involving patients in hematology wards (*4*,*16*); *S. clavata* infections are less often reported but occur as sporadic cases or small outbreaks that are usually (*2*,*10*,*11*), but not always (*1*), monocentric. 

No study of *S. clavata* outbreaks has so far succeeded in identifying the contamination source (*10*). Contaminated milk jugs have been identified as the source of outbreaks from *M. capitatus* (*17*), and several reports have noted the role of food as a potential source of outbreaks of *Geotrichum* spp. (*17*,*18*). However, because of the lack of accurate databases, earlier reports relied on the association of arthroconidia with lack of urease activity to identify *Geotrichum* spp., and others misidentified *S. clavata* as *M. capitatus* (*19*). Therefore, it is possible that cheese and milk that were reported in the literature (*5*,*17*) to be positive with *Geotrichum* spp. could actually have been contaminated by *S. clavata*. However, to our knowledge, no report has associated *S. clavata* with cheese production (*20*). 

Previously, we discovered that some yeast strains recovered from dishwashers were *S. clavata* and not *M. capitatus* as initially reported (*1*,*6*), which might reinforce ingestion as a possible route of *S. clavata* infection. This finding influenced our decision to sample dishwashers and the jugs and vacuum flasks used to deliver food to patients in the hematology and stem cell transplant wards at the cancer center. Recovering *S. clavata* from the dishwashers and jugs was the first step in explaining this monocentric outbreak, because the contaminated utensils from the hematology ward had been washed in those dishwashers. Another possible factor in the dishwasher’s involvement in spreading infection might have been the nonremovable lids on the jugs, which could have prevented the dishwasher from completely removing food residues. In a laboratory setting, *S. clavata* has been shown to not survive temperatures >48°C or contact with fungicidal sprays (M. Desnos-Ollivier, unpub. data). Therefore, it is possible that the temperature cycle of the dishwasher, normally capable of reaching temperatures >60°C, may have been dysregulated or the procedure or the detergent used to decontaminate dishes and utensils may have been insufficient. We did not assess these possibilities, but discarding the contaminated fomites and the old dishwasher seemed to control the outbreak. The dishwasher in the central kitchen was also contaminated, but only in the prewash area, ruling out its involvement in the spread of the fungus. Nevertheless, it was decontaminated as a precaution. Finally, we did not uncover any food source for the *S. clavata* infection, possibly because the initial contamination had occurred almost 2 years earlier or because we did not test the correct food samples. 

The temporal association of *S. clavata* in the environment with the outbreak offered only a potential link; genetic relatedness needed to be demonstrated. WGS is being used increasingly to investigate outbreaks, especially when genotyping methods are not readily available, such as for rare species. In 2012, following the discovery of a clade, A, as the source of a multicenter outbreak of *S. clavata* infections in France, we designed a real-time PCR so we could rapidly distinguish isolates belonging to clade A or to another clade, B (NRCMA, unpub. data). Since the isolates recovered in Marseille belonged to neither of those clades, we used WGS to study strain relatedness. All of the isolates recovered in Marseille after early 2016, including environmental isolates, clustered together into a novel clade, C, different from the previously identified clades. Identifying a unique clade suggested a common source for the contamination, which was restricted to this cancer center in Marseille. Of note, a single case diagnosed in the cancer center in 2015 did not belong to clades A, B, or C and was thus considered a sporadic case. 

Our investigation found that a dishwasher made available to patients in the kitchen of the stem cell transplant ward had been the vector of contamination. The fact that patient 4 had been hospitalized in neither the stem cell transplant ward nor the hematology ward before being infected leaves open the hypothesis that contaminated food, of an unknown source, could have contaminated utensils and then the dishwashers, which became vectors of *S. clavata* for other patients. This transmission scheme is supportable using our findings: the contaminated milk or coffee pitchers were used in both hematology and stem cell transplant units; environmental and clinical isolates clustered within the same clade; and the outbreak ended after we removed the pitchers, replaced the contaminated and potentially dysfunctional dishwasher in the stem cell transplant ward, and disinfected the dishwasher in the central kitchen. 

Our findings suggest that food-related household appliances, such as dishwashers, can be anthropophilic ecologic niches for *S. clavata* and other life-threatening fungi. Combined with the trend toward providing patients a low-bacterial diet rather than a sterile diet (*21*), this possibility increases the potential for contaminated food. Therefore, routine procedures to protect severely ill patients from airborne or contact contamination should include regular microbiologic sampling, dishwasher testing and maintenance, and controlling the supply and distribution of food. In general, these findings stress the need for continuous extensive vigilance in hospital settings.

## Acknowledgments

Appendix Additional information about *Saprochaete clavata* outbreak infecting cancer center through dishwasher.

## References

[1] Vaux S, Criscuolo A, Desnos-Ollivier M, Diancourt L, Tarnaud C, Vandenbogaert M, et al.; Geotrichum Investigation Group. Multicenter outbreak of infections by *Saprochaete clavata*, an unrecognized opportunistic fungal pathogen. MBio. 2014;5:e02309-14. 10.1128/mBio.02309-14 25516620 PMC4271555

[2] Del Principe MI, Sarmati L, Cefalo M, Fontana C, De Santis G, Buccisano F, et al. A cluster of *Geotrichum clavatum* (*Saprochaete clavata*) infection in haematological patients: a first Italian report and review of literature. Mycoses. 2016;59:594–601. 10.1111/myc.12508 27061932

[3] Bouza E, Muñoz P. Invasive infections caused by *Blastoschizomyces capitatus* and *Scedosporium* spp. Clin Microbiol Infect. 2004;10(Suppl 1):76–85. 10.1111/j.1470-9465.2004.00842.x 14748804

[4] García-Ruiz JC, López-Soria L, Olazábal I, Amutio E, Arrieta-Aguirre I, Velasco-Benito V, et al. Invasive infections caused by *Saprochaete capitata* in patients with haematological malignancies: report of five cases and review of the antifungal therapy. Rev Iberoam Micol. 2013;30:248–55. 10.1016/j.riam.2013.02.004 23583265

[5] Bouakline A, Lacroix C, Roux N, Gangneux JP, Derouin F. Fungal contamination of food in hematology units. J Clin Microbiol. 2000;38:4272–3. 10.1128/JCM.38.11.4272-4273.2000 11060109 PMC87582

[6] Zalar P, Novak M, de Hoog GS, Gunde-Cimerman N. Dishwashers—a man-made ecological niche accommodating human opportunistic fungal pathogens. Fungal Biol. 2011;115:997–1007. 10.1016/j.funbio.2011.04.007 21944212

[7] Girmenia C, Pagano L, Martino B, D’Antonio D, Fanci R, Specchia G, et al.; GIMEMA Infection Program. Invasive infections caused by *Trichosporon* species and *Geotrichum* capitatum in patients with hematological malignancies: a retrospective multicenter study from Italy and review of the literature. J Clin Microbiol. 2005;43:1818–28. 10.1128/JCM.43.4.1818-1828.2005 15815003 PMC1081342

[8] Bretagne S, Renaudat C, Desnos-Ollivier M, Sitbon K, Lortholary O, Dromer F; French Mycosis Study Group. Predisposing factors and outcome of uncommon yeast species-related fungaemia based on an exhaustive surveillance programme (2002-14). J Antimicrob Chemother. 2017;72:1784–93. 10.1093/jac/dkx045 28333259

[9] Arendrup MC, Boekhout T, Akova M, Meis JF, Cornely OA, Lortholary O; European Society of Clinical Microbiology and Infectious Diseases Fungal Infection Study Group; European Confederation of Medical Mycology. ESCMID and ECMM joint clinical guidelines for the diagnosis and management of rare invasive yeast infections. Clin Microbiol Infect. 2014;20(Suppl 3):76–98. 10.1111/1469-0691.12360 24102785

[10] Stanzani M, Cricca M, Sassi C, Sutto E, De Cicco G, Bonifazi F, et al. *Saprochaete clavata* infections in patients undergoing treatment for haematological malignancies: A report of a monocentric outbreak and review of the literature. Mycoses. 2019;62:1100–7. 10.1111/myc.12978 31365161

[11] Buchta V, Bolehovská R, Hovorková E, Cornely OA, Seidel D, Žák P. *Saprochaete clavata* invasive infections – a new threat to hematological-oncological patients. Front Microbiol. 2019;10:2196. 10.3389/fmicb.2019.02196 31736883 PMC6830389

[12] Durán Graeff L, Seidel D, Vehreschild MJGT, Hamprecht A, Kindo A, Racil Z, et al.; FungiScope Group. Invasive infections due to *Saprochaete* and *Geotrichum* species: Report of 23 cases from the FungiScope Registry. Mycoses. 2017;60:273–9. 10.1111/myc.12595 28150341

[13] Cassagne C, Normand A-C, Bonzon L, L’Ollivier C, Gautier M, Jeddi F, et al. Routine identification and mixed species detection in 6,192 clinical yeast isolates. Med Mycol. 2016;54:256–65. 10.1093/mmy/myv095 26613703

[14] Li H. Minimap2: pairwise alignment for nucleotide sequences. Bioinformatics. 2018;34:3094–100. 10.1093/bioinformatics/bty191 29750242 PMC6137996

[15] Nguyen L-T, Schmidt HA, von Haeseler A, Minh BQ. IQ-TREE: a fast and effective stochastic algorithm for estimating maximum-likelihood phylogenies. Mol Biol Evol. 2015;32:268–74. 10.1093/molbev/msu300 25371430 PMC4271533

[16] Trabelsi H, Néji S, Gargouri L, Sellami H, Guidara R, Cheikhrouhou F, et al. *Geotrichum capitatum* septicemia: case report and review of the literature. Mycopathologia. 2015;179:465–9. 10.1007/s11046-015-9869-2 25681053

[17] Gurgui M, Sanchez F, March F, Lopez-Contreras J, Martino R, Cotura A, et al. Nosocomial outbreak of *Blastoschizomyces capitatus* associated with contaminated milk in a haematological unit. J Hosp Infect. 2011;78:274–8. 10.1016/j.jhin.2011.01.027 21658800

[18] Benedict K, Chiller TM, Mody RK. Invasive fungal infections acquired from contaminated food or nutritional supplements: a review of the literature. Foodborne Pathog Dis. 2016;13:343–9. 10.1089/fpd.2015.2108 27074753 PMC5669373

[19] Desnos-Ollivier M, Blanc C, Garcia-Hermoso D, Hoinard D, Alanio A, Dromer F. Misidentification of *Saprochaete clavata* as *Magnusiomyces capitatus* in clinical isolates: utility of internal transcribed spacer sequencing and matrix-assisted laser desorption ionization-time of flight mass spectrometry and importance of reliable databases. J Clin Microbiol. 2014;52:2196–8. 10.1128/JCM.00039-14 24696028 PMC4042774

[20] Fröhlich-Wyder M-T, Arias-Roth E, Jakob E. Cheese yeasts. Yeast. 2019;36:129–41. 10.1002/yea.3368 30512214

[21] van Dalen EC, Mank A, Leclercq E, Mulder RL, Davies M, Kersten MJ, et al. Low bacterial diet versus control diet to prevent infection in cancer patients treated with chemotherapy causing episodes of neutropenia. Cochrane Database Syst Rev. 2016;4:CD006247. 10.1002/14651858.CD006247.pub3 27107610 PMC6466670

