# Supplementary material for: Saprochaete clavata Outbreak Infecting Cancer Center through Dishwasher
Source: Emerg Infect Dis. 2020 Sep;26(9):2031–8. doi: 10.3201/eid2609.200341 (PMC7454083; doi:10.3201/eid2609.200341)
Supplement: Appendix — Additional information about Saprochaete clavata outbreak infecting cancer center through dishwasher. [file 20-0341-Techapp-s1.pdf]

Article DOI: https://doi.org/10.3201/eid2609.200341

Outbreak of *Saprochaete clavata* Infections in Cancer Center through Dishwasher

Appendix

Appendix Table 1. Isolates for which whole genome was sequenced*

Strain	Location	Patient no.	Site isolated from	Date isolated	Accession number
CNRMA16.170	Marseille cancer ctr	1	Blood	03/02/2016	SAMEA6496805
CNRMA17.141	Marseille cancer ctr	2	Blood	16/01/2017	SAMEA6496813
CNRMA18.130	Marseille cancer ctr	2	Blood	16/01/2017	SAMEA6496812
CNRMA18.131	Marseille cancer ctr	3	Trachea	18/01/2017	SAMEA6496816
CNRMA18.134	Marseille cancer ctr	4	Blood	28/02/2017	SAMEA6496804
CNRMA18.10	Marseille cancer ctr	5	Blood	17/04/2017	SAMEA6496815
CNRMA17.313	Marseille cancer ctr	5	Blood	17/04/2017	SAMEA6496814
CNRMA18.137	Marseille cancer ctr	6	Blood	30/06/2017	SAMEA6496807
CNRMA18.9	Marseille cancer ctr	7	Blood	06/12/2017	SAMEA6496817
CNRMA18.8	Marseille cancer ctr	8	Blood	10/12/2017	SAMEA6496824
CNRMA18.35	Marseille cancer ctr	8	Blood	03/01/2018	SAMEA6496827
CNRMA18.31	Marseille cancer ctr	8	Blood	14/12/2017	SAMEA6496825
CNRMA18.32	Marseille cancer ctr	8	Blood	27/12/2017	SAMEA6496826
CNRMA18.34	Marseille cancer ctr	9	Blood	01/01/2018	SAMEA6496809
CNRMA18.36	Marseille cancer ctr	9	Blood	18/01/2018	SAMEA6496810
CNRMA18.11	Marseille cancer ctr	9	Blood	29/12/2017	SAMEA6496808
CNRMA12.494	Marseille cancer ctr	10	Blood	23/01/2015	SAMEA6496801
CNRMA18.28	Marseille cancer ctr	NA	Prewash (central kitchen dishwasher)	04/01/2018	SAMEA6496802
CNRMA18.33	Marseille cancer ctr	NA	Water outlet (ward dishwasher)	27/12/2017	SAMEA6496803
CNRMA18.93	Marseille cancer ctr	NA	Coffee pitcher lid	04/01/2018	SAMEA6496806
CNRMA18.29	Marseille cancer ctr	NA	Milk pitcher lid	04/01/2018	SAMEA6496811
CNRMA18.26	Marseille cancer ctr	NA	Vacuum flask for coffee	26/12/2017	SAMEA6496822
CNRMA18.25	Marseille cancer ctr	NA	Surface, room patient 8	26/12/2017	SAMEA6496821
CNRMA18.24	Marseille cancer ctr	NA	Seal (ward dishwasher)	26/12/2017	SAMEA6496820
CNRMA18.27	Marseille cancer ctr	NA	Vacuum flask for milk	18/01/2018	SAMEA6496823
CNRMA18.23	Marseille cancer ctr	NA	Upper surface (ward dishwasher)	26/12/2017	SAMEA6496819
CNRMA18.22	Marseille cancer ctr	NA	Tank bottom (ward dishwasher)	26/12/2017	SAMEA6496818
CBS425.71	USA/Baltimore	NA	Lung tissue	1971	SAMEA2186846
CNRMA12.304	France/Hospital 1	11	Blood	20/03/2012	SAMEA2186850
CNRMA11.1183	France/Hospital 2	12	Blood	11/12/2011	SAMEA2186848
CNRMA8.1167	France/Hospital 3	13	Blood	08/09/2008	SAMEA2186871
CNRMA12.615	France/Hospital 4	14	Blood	20/10/2011	SAMEA2186867
CNRMA12.634	France/Hospital 5	15	Blood	26/06/2011	SAMEA2186853
CNRMA12.667	France/Hospital 6	16	Blood	22/06/2012	SAMEA2186869
CNRMA12.559	France/Hospital 7	17	Blood	07/05/2012	SAMEA2186866
CNRMA12.637	France/Hospital 5	18	Stool	29/05/2012	SAMEA2186854
CNRMA12.494	France/Hospital 8	19	Blood	11/05/2012	SAMEA2186855
CNRMA12.647	France/Hospital 2	20	Blood	09/05/2012	SAMEA2186855

***NA, not applicable.**
